# Comparative Genomics of *Burkholderia singularis* sp. nov., a Low G+C Content, Free-Living Bacterium That Defies Taxonomic Dissection of the Genus *Burkholderia*

**DOI:** 10.3389/fmicb.2017.01679

**Published:** 2017-09-06

**Authors:** Peter Vandamme, Charlotte Peeters, Birgit De Smet, Erin P. Price, Derek S. Sarovich, Deborah A. Henry, Trevor J. Hird, James E. A. Zlosnik, Mark Mayo, Jeffrey Warner, Anthony Baker, Bart J. Currie, Aurélien Carlier

**Affiliations:** ^1^Laboratory of Microbiology, Department of Biochemistry and Microbiology, Faculty of Sciences, Ghent University Ghent, Belgium; ^2^Global and Tropical Health Division, Menzies School of Health Research, Darwin NT, Australia; ^3^Centre for Animal Health Innovation, Faculty of Science, Health, Education and Engineering, University of the Sunshine Coast, Sippy Downs QLD, Australia; ^4^Centre for Understanding and Preventing Infection in Children, Department of Pediatrics, University of British Columbia, Vancouver BC, Canada; ^5^College of Public Health, Medical and Veterinary Sciences, Australian Institute of Tropical Health and Medicine, James Cook University, Townsville QLD, Australia; ^6^Tasmanian Institute of Agriculture, University of Tasmania, Hobart TAS, Australia

**Keywords:** *Burkholderia singularis*, whole genome sequence, cystic fibrosis microbiology, comparative genomics, *Burkholderia pseudomallei* complex, *Burkholderia cepacia* complex

## Abstract

Four *Burkholderia pseudomallei*-like isolates of human clinical origin were examined by a polyphasic taxonomic approach that included comparative whole genome analyses. The results demonstrated that these isolates represent a rare and unusual, novel *Burkholderia* species for which we propose the name *B. singularis.* The type strain is LMG 28154^T^ (=CCUG 65685^T^). Its genome sequence has an average mol% G+C content of 64.34%, which is considerably lower than that of other *Burkholderia* species. The reduced G+C content of strain LMG 28154^T^ was characterized by a genome wide AT bias that was not due to reduced GC-biased gene conversion or reductive genome evolution, but might have been caused by an altered DNA base excision repair pathway. *B. singularis* can be differentiated from other *Burkholderia* species by multilocus sequence analysis, MALDI-TOF mass spectrometry and a distinctive biochemical profile that includes the absence of nitrate reduction, a mucoid appearance on Columbia sheep blood agar, and a slowly positive oxidase reaction. Comparisons with publicly available whole genome sequences demonstrated that strain TSV85, an Australian water isolate, also represents the same species and therefore, to date, *B. singularis* has been recovered from human or environmental samples on three continents.

## Introduction

A variety of Gram-negative non-fermenting bacteria can colonize the lungs of patients with cystic fibrosis (CF) (Lipuma, 2010; Parkins and Floto, 2015). The majority of these bacteria are considered opportunists but correct species identification is paramount for the management of infections in these patients. More than twenty *Burkholderia* species have been retrieved from respiratory samples of patients with CF, with *B. cenocepacia* and *B. pseudomallei* the major concerns (O’Carroll et al., 2003; Lipuma, 2010). When the genus *Burkholderia* was created in 1992 it consisted of only seven species which were primarily known as human, animal, and plant pathogens (Yabuuchi et al., 1992). They proved, however, to be metabolically versatile and biotechnologically appealing, and a large number of novel *Burkholderia* species were subsequently isolated and formally named. The genus *Burkholderia* now comprises about 100 validly named species^1^ and many uncultivated candidate species, which occupy extremely diverse ecological niches (Coenye and Vandamme, 2003; Compant et al., 2008; Suarez-Moreno et al., 2012).

Although their metabolic potential can be exploited for biocontrol, bioremediation and plant growth promotion, *Burkholderia* species are notorious opportunistic pathogens in immunocompromised patients and cause pseudo-outbreaks through their impressive capacity to contaminate pharmaceutical products (Coenye and Vandamme, 2003; Torbeck et al., 2011; Depoorter et al., 2016). The use of *Burkholderia* in agricultural applications is therefore controversial and considerable effort has been invested to discriminate beneficial from clinical *Burkholderia* strains (Baldwin et al., 2007; Mahenthiralingam et al., 2008). Recently, the phylogenetic diversity within this genus, as revealed through comparative 16S ribosomal RNA (rRNA) gene studies, was used to subdivide this genus into *Burkholderia sensu stricto* (which comprises the majority of human pathogens) and several novel genera, i.e., *Paraburkholderia*, *Caballeronia*, and *Robbsia* (Sawana et al., 2014; Dobritsa and Samadpour, 2016; Lopes-Santos et al., 2017). This 16S rRNA sequence based subdivision was supported by a difference in genomic G+C content: *Burkholderia sensu stricto* species have a genomic G+C content of 65.7 to 68.5%, whereas the genera *Paraburkholderia*, *Caballeronia* and *Robbsia* have a genomic G+C content of about 61.4–65.0%, 58.9–65.0%, and 58.9%, respectively.

In our studies of the biodiversity of *Burkholderia* strains that are recovered from samples of CF patients, we received isolates from a Canadian and a German patient that proved difficult to identify using conventional phenotyping and genotyping methods. Although first considered *B. cepacia-*like bacteria, partial 16S rRNA gene sequencing revealed that they were more closely related to *B. pseudomallei*. The present study provides detailed genotypic and phenotypic characterization of this novel bacterium and shows that it has genomic signatures that defy the recent dissection of the genus *Burkholderia.*

## Materials and Methods

### Bacterial Strains and Growth Conditions

The isolates R-20802 (=VC11777), LMG 28154^T^ (=VC12093) and R-50762 (=VC15152) are serial isolates from respiratory samples of the same Canadian CF patient and were collected in March 2003, October 2003 and 2009, respectively (**Table 1**). Isolate LMG 28155 was obtained from a German CF patient in 2008. Strains were grown aerobically on Tryptone Soya Agar (Oxoid) and were routinely incubated at 28°C. Cultures were preserved in MicroBank^TM^ vials at -80°C until lyophilization.

**Table 1:** Isolates studied, their sources, sequence types and allelic profiles.

Strain^1^	Other strain designations	Source^2^	Depositor	ST^3^	Allelic profile						
					
		(Country, year of isolation)			*atpD*	*gltB*	*gyrB*	*recA*	*lepA*	*phaC*	*trpB*
LMG 28154^T^	VC11777^T^, CCUG 65685^T^	CF (Canada, 2003)	Current study	813	337	391	583	355	407	311	393
R-20802	VC12093	CF (Canada, 2003)	Current study	813	337	391	583	355	407	311	393
R-50762	VC15152	CF (Canada, 2009)	Current study	813	337	391	583	355	407	311	393
LMG 28155	848157/5638	CF (Germany, 2008)	L. Sedlacek	815	338	392	584	356	408	311	394
TSV85		Water (Australia, 2010)	Current study	1295	404	564	848	481	524	311	524


*^1^LMG, BCCM/LMG Bacteria Collection, Laboratory of Microbiology, Ghent University, Ghent, Belgium; CCUG, Culture Collection University of Gothenburg, Department of Clinical Bacteriology, Sahlgrenska University Hospital, Gothenburg, Sweden.*

*^2^CF, isolated from a cystic fibrosis patient.*

*^3^Based on the *B. cepacia* complex scheme (https://pubmlst.org/bcc/).*

### 16S rRNA Gene Sequence Analysis

The 16S rRNA gene sequences of strains LMG 28154^T^ and LMG 28155 were determined as described by Vandamme et al. (2007). Sequence assembly was performed using the BioNumerics v7 software. EzBioCloud was used to identify the nearest neighbor taxa with validly published names (Yoon et al., 2017). The 16S rRNA sequences of two strains without taxonomic standing, i.e., *Burkholderia* sp. TSV85 (GenBank accession GCA_001523725.1) and *Burkholderia* sp. TSV86 (GenBank accession GCA_001522865.1), were retrieved from their whole genome sequences as preliminary analysis of genome sequences indicated a close phylogenetic relationship to strain LMG 28154^T^. The 16S rRNA sequences of strains LMG 28154^T^, LMG 28155, TSV85, TSV86 and *Burkholderia* representatives (1011–1610 bp) were aligned against the SILVA SSU reference database using SINA v1.2.11^2^ (Pruesse et al., 2012). Phylogenetic analysis was conducted using MEGA7 (Kumar et al., 2016). All positions with <95% site coverage were eliminated, resulting in a total of 1311 positions in the final dataset. The statistical reliability of tree topologies was evaluated by bootstrapping analysis based on 1000 replicates.

### MLST Analysis

MLST analysis was based on the method described by Spilker et al. (2009) with small modifications as described earlier (Peeters et al., 2013). Nucleotide sequences of each allele, allelic profiles and sequence types for all isolates from the present study are available on the *B. cepacia* complex PubMLST website^3^ (Jolley et al., 2004; Jolley and Maiden, 2010). Although most closely related to *B. pseudomallei* complex species according to 16S rRNA and whole genome phylogenies, strains LMG 28154^T^, TSV85 and TSV86 were not able to be genotyped with the *B. pseudomallei* PubMLST scheme^4^ due to the absence of the *narK* locus, consistent with other non-*B. pseudomallei* complex species (Price et al., 2016).

### Genome Sequencing, Assembly and Annotation

Strain LMG 28154^T^ was grown for 48 h on chocolate agar (Oxoid) and genomic DNA was prepared by the CTAB and phenol:chloroform purification method (Currie et al., 2007). Paired-end 100 bp libraries were sequenced on an Illumina HiSeq2000 sequencer (Macrogen Inc., Geumcheon-gu, Seoul, South Korea). Sequencing reads were quality-filtered and assembled as described earlier (Carlier et al., 2016). Briefly, the reads were prepared for assembly using the adapter trimming function of the Trimmomatic software (Bolger et al., 2014) and sequencing reads with a Phred score below 20 were discarded. The trimmed reads were used for *de novo* assembly using SPAdes v3.0 assembler with k-mer lengths of 21, 33, 55, 77, and 89 (Bankevich et al., 2012). The QUAST program was used to generate the summary statistics of the assembly (N50, maximum contig length, GC) (Gurevich et al., 2013). Trimmed reads were mapped back to the assembled contigs using the SMALT software^5^ and mapping information was extracted using the Samtools software suite. Contigs <500 bp and with <35× coverage were discarded. Final contigs were checked for contamination against common contaminants (e.g., PhiX 174 genome sequence) and submitted for annotation to the RAST online service (Aziz et al., 2008) with gene prediction option enabled. Annotated contig sequences were manually curated in the Artemis software (Carver et al., 2008). Annotated contigs were deposited in the European Nucleotide Archive^6^ with the accession numbers FXAN01000001–FXAN01000135. Raw sequencing reads are available under accession ERR1923794.

Ortholog computations were done with the Orthomcl v1.4 software (Li et al., 2003), using the NCBI Blastp software (Altschul et al., 1997) with *e*-value cut-off of 1.0 × 10^-6^ and a percentage identity cut-off of 50%. Assignment of Clusters of Orthologous Genes (COG) functional categories was done by searching protein sequences in the COG database (Galperin et al., 2015) using the NCBI rpsblast software with an *e*-value cut-off of 10^-3^. COG accessions were searched in the STRING v10 database (von Mering et al., 2005) and interaction networks were drawn by selecting neighborhood and co-expression interaction sources. Genes belonging to each COG category were counted and the distributions were compared using a Pearson’s chi-squared test run with the R software package (R Core Team, 2014).

Putative pseudogenes were predicted as described earlier (Pinto-Carbo et al., 2016). Briefly, predicted genes were searched against a database of *Burkholderia* orthologs calculated as described above and intergenic regions using BLASTx (Altschul et al., 1990) against a custom database of protein sequences predicted from *Burkholderia* genomes. Only hits with >50% identity and with an *e*-value < 10^-6^ were considered. Hit regions were aligned with the best-scoring subject protein sequence using the tfasty program of the FASTA software suite v3.6 in order to find the boundaries of the pseudogenes (Pearson, 2000). Hits with <80% of the ORF length intact (either due to frameshift or early stop codon) were considered putative pseudogenes.

### Average Nucleotide Identity (ANI) Values

Calculation of ANI values was done on a whole genome sequence database of *Burkholderia* species (type strains if genome sequence was available at the time of analysis, otherwise representative genome sequence as listed by NCBI GenBank): *B. thailandensis* E264^T^ (GenBank accession GCA_000012365.1), *B. pseudomallei* K96243 (GenBank accession GCA_000011545.1), *B. oklahomensis* C6786^T^ (GenBank accession GCA_000170375.1), *B. mallei* ATCC 23344^T^ (GenBank accession GCA_000011705.1), *B. humptydooensis* MSMB43^T^ (GenBank accession GCA_001513745.1), *Burkholderia* sp. TSV85 (GenBank accession GCA_001523725.1) and *Burkholderia* sp. TSV86 (GenBank accession GCA_001522865.1). The draft assembly data of strain LMG 28154^T^ in FASTA format was uploaded to the JSpeciesWS website (Richter and Rossello-Mora, 2009). ANI analysis was performed with the ANIb algorithm (Goris et al., 2007) accessible via the JSpeciesWS web service (Richter and Rossello-Mora, 2009).

### Whole Genome Phylogeny

Representative genomes from individual *Burkholderia*, *Paraburkholderia*, *Caballeronia* and *Robbsia* species were downloaded from the NCBI RefSeq database (accessed Feb. 2017). In addition, genomes from strains LMG 28154^T^, TSV85 and TSV86 were added to the database. Nucleotide and amino acid sequences of annotated CDS were extracted and searched against a database of 40 nearly universal, single copy gene markers using the FetchMG program with the –v flag to retain only the best hits (Sunagawa et al., 2013). Twenty-three COGs present in all genomes were extracted, aligned with Clustal Omega and the resulting alignments were concatenated using the AlignIO utility of Biopython (Cock et al., 2009). The concatenated alignment was trimmed with TrimAl (Capella-Gutierrez et al., 2009) to remove columns with >10% gaps and poorly aligned sections of the alignment were removed manually in CLC Main Workbench v.7.7 (Qiagen, Aarhus, Denmark). A concatenated nucleotide alignment of 15092 bp was used to build a phylogenetic tree with FastTree, using the GTR CAT model (Price et al., 2010). The tree was edited in iTOL (Letunic and Bork, 2016).

### G+C Content and Evolutionary Rates Analysis

Overall mol% G+C was calculated from the final set of genome contigs using the Quast software (Gurevich et al., 2013). Intra-genome %G+C distributions were calculated on non-overlapping 1-kb fragments using *ad hoc* Python scripts. Extreme values were discarded for clarity, and the median and quartile values, as well as the estimated size of the genomes were plotted together with the phylogenetic tree using the iTol v3 web server (Letunic and Bork, 2016). Putative orthologs between the genomes of strain LMG 28154^T^, *B. thailandensis* E264^T^, *B. pseudomallei* K96243, *B. oklahomensis* C6786^T^, *Burkholderia* sp. TSV86 and *B. glumae* BGR1 (Genbank accession GCA_000022645.2) were calculated using the Orthomcl software as described above. Single copy orthologs from each genome were selected and the protein sequences were aligned using MUSCLE with standard settings (Edgar, 2004) and back-translated into nucleotide alignments using T-Coffee (Notredame et al., 2000). Poorly aligned regions and gaps were trimmed using the TrimAl software (Capella-Gutierrez et al., 2009) and overall G+C content as well as G+C content for each codon position were calculated using *ad hoc* Python 2.7.3 scripts (scripts available upon request). Statistical tests were performed with the R software package version 3.1.0 (R Core Team, 2014), including the pgirmess package for non-parametric Kruskal–Wallis tests (Giraudoux, 2013). Statistical tests for recombination were conducted on the trimmed nucleotide alignments for each single copy orthologous groups using the PhiPack software (Bruen et al., 2006) and the *p*-values were calculated with 1000 permutations and a 100 bp window. Alignments which failed the permutation test were discarded and a *p*-value < 0.05 was considered as evidence of recombination. Only orthologous groups without statistical evidence of recombination (*p*-value > 0.05) were used for the calculation of evolutionary rates.

Rates of synonymous (*dS*) and non-synonymous (*dN*) substitutions were estimated for the gene families with strictly one ortholog in each of the eight species. Protein sequences for each ortholog cluster were aligned with MUSCLE and subsequent nucleotide codon alignments were generated and trimmed using Pal2nal perl script (Suyama et al., 2006).

Pairwise *dN*/*dS* values were estimated with the yn00 module of the PAML v4.4 package (Yang, 2007) using the Yang and Nielsen (2000) method. Pairs of orthologs with insufficient levels of divergence (*dS* < 0.1) or near saturation (*dS* > 1) were excluded from the analyses. Pairwise ratios of non-synonymous to synonymous (*dN*/*dS*) mutations were extracted from the PAML output using *ad hoc* Python scripts and plotted in R. Non-parametric Wilcoxon signed rank tests were performed to test whether *dN*/*dS* values were higher for genes in the novel species compared to free-living *Burkholderia* (null hypothesis).

Codon frequencies were calculated on sets of orthologous genes that did not show evidence of recombination (*n* = 2890) with the Cusp program of the Emboss toolkit (Rice et al., 2000). Only fourfold degenerate codons were considered for analysis. Statistical analyses were done in R.

For the calculation of substitution bias in intergenic regions, we selected a random set of 17 intergenic regions which were conserved in strains LMG 28154^T^, TSV85 and TSV86, and did not contain mis-annotated CDS as evaluated by BLASTx against the NCBI nr database (accessed March 2017). Sequences were aligned using MUSCLE and the alignments were visualized in the CLC Main Workbench v.7 software (Qiagen, Aarhus, Denmark). Only substitutions for which the ancestral state could be determined unambiguously (conserved in at least 2 sequences, including in the *Burkholderia* sp. TSV86 genome) were considered.

### Real-time PCR Assays

Real-time PCR assays were performed as described earlier (Novak et al., 2006; Price et al., 2012).

### Fatty Acid Methyl Ester Analysis

After a 24 h incubation period at 28°C on Tryptone Soya Agar (BD), a loopful of well-grown cells was harvested and fatty acid methyl esters were prepared, separated and identified using the Microbial Identification System (Microbial ID) as described previously (Vandamme et al., 1992).

### Biochemical Characterization

Biochemical characterization was performed as described previously (Henry et al., 2001).

### MALDI-TOF Mass Spectrometry

MALDI-TOF MS was performed as described previously (De Bel et al., 2011) using a Microflex LT MALDI-TOF MS instrument (flexControl version 3.4, MALDI Biotyper Compass Explorer 4.1) with the reference database version 6.0.0.0 (6,903 database entries) (Bruker Daltonik).

## Results and Discussion

### Misidentification of Strain LMG 28154^T^

The identification of Gram-negative non-fermenting bacteria isolated from respiratory secretions of people with CF may prove challenging, not the least because the CF lung can harbor a range of opportunistic bacteria rarely seen in the general population. Many of these opportunists represent novel species whose correct diagnosis requires formal description. In the present study, we report genomic and basic taxonomic characteristics for the formal description of a rare but most unusual *Burkholderia* species that can colonize the CF lung. Preliminary biochemical characterization, including the observation of a typical slowly positive oxidase reaction, suggested this organism was a *B. cepacia* complex bacterium, yet amplification of the *recA* gene (Mahenthiralingam et al., 2000), a standard approach for the identification of *B. cepacia* complex bacteria, proved repeatedly negative (data not shown). Also, colonies of each of these isolates had a mucoid appearance on Columbia sheep’s blood agar, a characteristic that is rarely seen among *B. cepacia* complex bacteria. MALDI-TOF MS analysis yielded *B. multivorans* as best hit for each of the isolates. Score values of cell smears ranged between 1.66 and 1.87, with a difference of less than 0.2 toward the next species, suggesting this was a *Burkholderia*, but not providing species level identification (De Bel et al., 2011). Similarly, score values of cell extracts ranged between 1.61 and 1.96, again with a difference of less than 0.2 toward the next species. Initial partial 16S rRNA gene sequence analysis unexpectedly yielded *B. pseudomallei* and relatives as nearest neighbor species, rather than *B. cepacia* complex bacteria. While these isolates indeed were arginine dihydrolase positive, like *B. pseudomallei* and *B. thailandensis*, they did not reduce nitrate. The 266152 and type III secretion system based real-time PCR assays (Novak et al., 2006; Price et al., 2012) were negative, which excluded their identification as *B. pseudomallei*, *B. thailandensis*, *B. humptydooensis* or *B. oklahomensis* (data not shown). Adding to the confusion of this diagnostic problem, were the unusual dry sheen overlaying a slightly mucoid colony texture in one patient’s first culture (R-20802), while a subsequent culture (LMG 28154^T^) was as mucoid as commonly observed for *Pseudomonas aeruginosa* isolates. In addition, the former culture looked mixed with small mucoid colonies and large mucoid colonies that exhibited different appearances but that yielded identical RAPD patterns (data not shown). This phenomenon, known as phenotypic switching, is commonly seen in pathogenic *Burkholderia* species (Chantratita et al., 2007; Bernier et al., 2008).

### Strain LMG 28154^T^ Represents a Novel Species in the *B. pseudomallei* Complex

**FIGURE 1:**
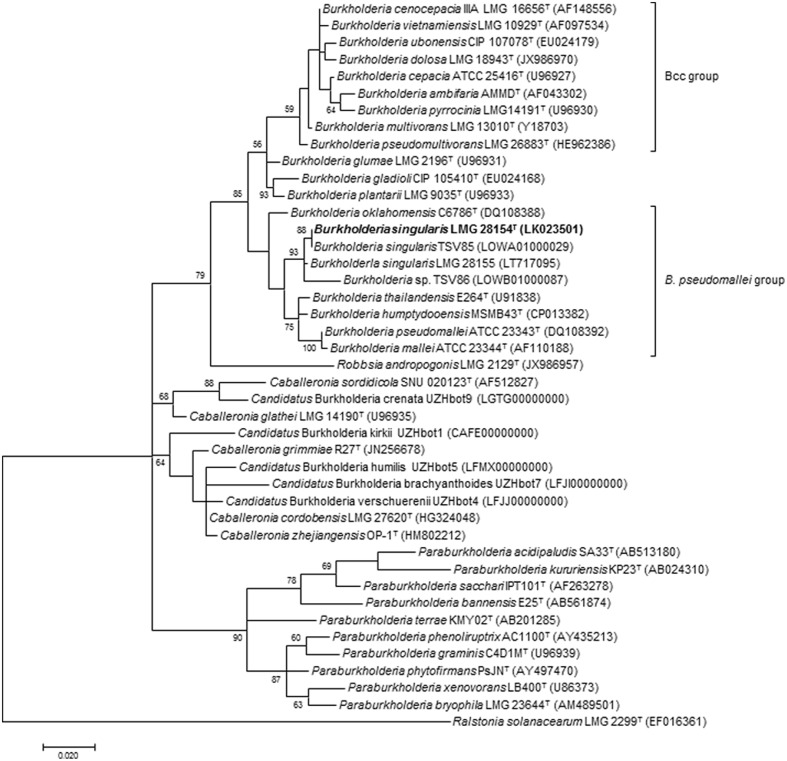
Phylogenetic tree based on nearly complete 16S rRNA gene sequences of *Burkholderia* representatives and *B. singularis* sp. nov. isolates. The optimal tree (highest log likelihood) was constructed using the Maximum Likelihood method and Tamura-Nei model in MEGA7 (Kumar et al., 2016). A discrete Gamma distribution was used to model evolutionary rate differences among sites [5 categories (+G, parameter = 0.3465)] and allowed for some sites to be evolutionarily invariable ([+I], 42.1434% sites). The percentage of replicate trees in which the associated taxa clustered together in the bootstrap test (1000 replicates) are shown next to the branches if higher than 50%. The sequence of *Ralstonia solanacearum* LMG 2299^T^ was used as outgroup. The scale bar indicates the number of substitutions per site. Taxonomic type strains are indicated by superscript T in strain numbers.

**FIGURE 2:**
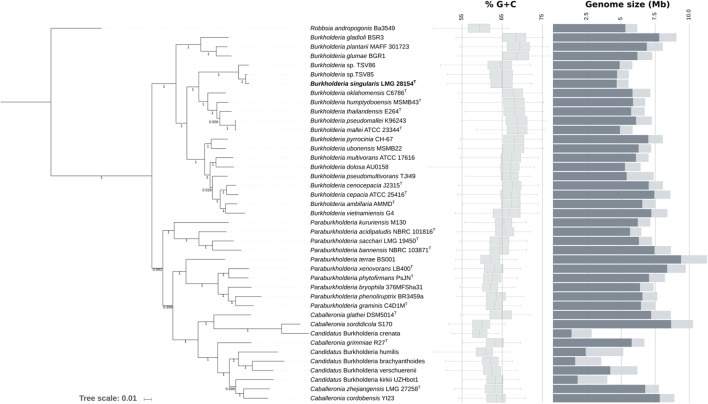
Genomic phylogeny and characteristics of the genus *Burkholderia*. The phylogenetic tree was built using an alignment of 23 conserved single copy COGs and an approximate maximum-likelihood approach (see Materials and Methods for details). The %G+C distribution was calculated on non-overlapping 1 kb segments for each genome and plotted as box plots with extreme values removed for clarity. Total genome size (bar plot) is approximated by size of the assembly and coding content (dark gray) corresponds to the sum of the length of all annotated CDS features, discounting predicted pseudogenes. Shimodaira-Hasegawa-like local support values as given by the program Fasttree are indicated.

To clarify the taxonomic status of these isolates, the nearly complete 16S rRNA gene sequences of strains LMG 28154^T^ and LMG 28155 were determined and proved nearly identical (99.3% pairwise identity). The similarity level of the 16S rRNA gene of strain LMG 28154^T^ toward those of the type strains to the nearest phylogenetic neighbors was 98.97% (*B. thailandensis*), 98.55% (*B. pseudomallei*), 98.49% (*B. mallei*), 98.60% (*B. humptydooensis*) and 98.35% (*B. oklahomensis*) (**Figure 1**); type strains of *B. cepacia* complex species had ≤98.32% 16S rRNA gene sequence identity. A draft whole genome sequence of strain LMG 28154^T^ was subsequently generated to further determine the degree of genomic relatedness of this novel bacterium with its nearest neighbor species. Genome assembly of strain LMG 28154^T^ yielded 135 contigs and a total assembly size of 5.54 Mb, with an N50 of 83 kb and an average coverage of 70×. Annotation revealed 5268 CDS and 50 tRNAs coding for all amino acids. The rRNA operon was assembled into a single contig due to the use of short-span read libraries that prohibit the resolution of larger repeats, but coverage analysis indicated the presence of four copies of the rRNA operon in the genome consistent with the rRNA operon copy number in *B. pseudomallei*, *B. thailandensis*, *B. humptydooensis* and *B. oklahomensis*. The 5268 predicted CDS features had an average length of 894 bp and a coding density of 85%, which was within the normal range for free-living *Burkholderia* species (**Figure 2**). ANI values between strain LMG 28154^T^ and its nearest phylogenetic neighbor species were below 85% (**Supplementary Table S1**), i.e., well below the commonly accepted threshold of 95–96% for species delineation (Richter and Rossello-Mora, 2009), confirming that strain LMG 28154^T^ represents a novel *Burkholderia* species. Remarkably, ANI values between strain LMG 28154^T^ and two water isolates (i.e., *Burkholderia* sp. TSV85 and *Burkholderia* sp. TSV86), whose whole genome sequences are publicly available, were 98.09 and 94.40, respectively, indicating that the former represents the same species. The water isolates were collected in 2010 from a single site (19°15′27.44″S; 146°47′36.20″E) in North Queensland, Australia, in a region endemic for *B. pseudomallei.* The ANI value of 94.40 for the latter strain is situated just below the species delineation threshold (Richter and Rossello-Mora, 2009), suggesting it represents a distinct, yet closely related novel *Burkholderia* species as revealed by its position in the 16S rRNA based phylogenetic tree (**Figure 1**). A phylogenetic analysis based on 15092 conserved nucleotide positions was subsequently performed to better reflect organismal phylogeny (**Figure 2**). This tree confirmed that the novel species represented by strain LMG 28154^T^ occupies a very distinct position within the *B. pseudomallei* complex of the genus *Burkholderia* (Vandamme and Peeters, 2014; Price et al., 2016) and that *Burkholderia* sp. TSV86 is closely related to it.

Standard biochemical identification of *B. pseudomallei* complex species is notoriously difficult (Glass et al., 2006). Yet, the present novel species can be distinguished from species in the *B. pseudomallei* complex through the absence of nitrate reduction and a mucoid appearance on Columbia sheep blood agar. In addition, its oxidase activity is typically slow as observed in *B. cepacia* complex species (Henry et al., 2001).

### Functional Content of the LMG 28154^T^ Genome

**FIGURE 3:**
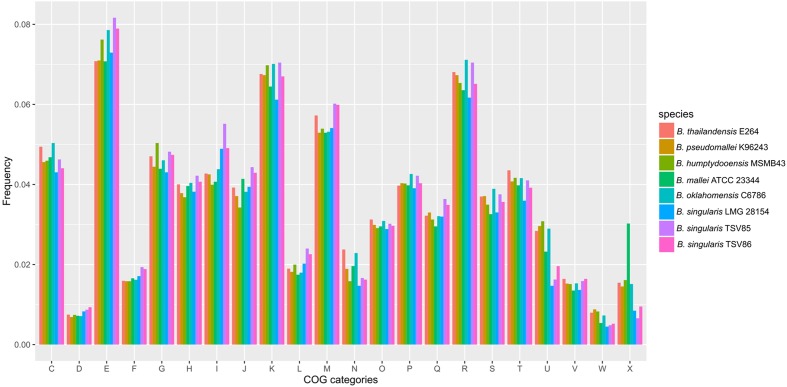
Distribution of genes in COG functional categories. Frequencies of genes in COG categories of selected *Burkholderia* genomes. The values represent the number of genes in COG categories, divided by the total number of genes with a COG assignment (genes without COG assignments are not counted for clarity). (C) = Energy production and conversion, (D) = Cell cycle control, cell division, chromosome partitioning, (E) = Amino acid transport and metabolism, (F) = Nucleotide transport and metabolism, (G) = Carbohydrate transport and metabolism, (H) = Coenzyme transport and metabolism, (I) = Lipid transport and metabolism, (J) = Translation, ribosomal structure and biogenesis, (K) = Transcription, (L) = Replication, recombination and repair, (M) = Cell wall/membrane/envelope biogenesis, (N) = Cell motility, (O) = Posttranslational modification, protein turnover, chaperones, (P) = Inorganic ion transport and metabolism, (Q) = Secondary metabolites biosynthesis, transport and catabolism, (R) = General function prediction only, (S) = Function unknown, (T) = Signal transduction mechanisms, (U) = Intracellular trafficking, secretion, and vesicular transport, (V) = Defense mechanisms, (W) = Extracellular structures, (X) = mobilome.

We compared the functional content of the LMG 28154^T^ genome to that of closely related *Burkholderia* species in order to find differences in gene content related to lifestyle or pathogenicity. We compared the distribution of genes assigned to each major COG functional category for strains LMG 28154^T^, *B. thailandensis* E264^T^, *B. pseudomallei* K96243 and *B. oklahomensis* C6786^T^ (**Figure 3**). The total number of genes with COG assignments was lower for strain LMG 28154^T^ than for the other strains (4241 vs. 5019–5445), owing to an overall smaller genome. However, the functional profile of strain LMG 28154^T^ was not significantly different from any of the other species (Pearson’s Chi squared *p*-value > 0.05), indicating that no specific functional category is enriched or reduced in strain LMG 28154^T^. Similarly, of the 5268 predicted CDS in the LMG 28154^T^ genome, 3248 are part of the core genome of strains LMG 28154^T^, *B. thailandensis* E264^T^, *B. pseudomallei* K96243 and *B. oklahomensis* C6786^T^, with only 1668 genes without predicted orthologs in the other three genomes. Further analyses revealed that the core genome of strains *B. thailandensis* E264^T^, *B. pseudomallei* K96243 and *B. oklahomensis* C6786^T^ possessed a dissimilatory nitrate reductase (*BPSL2308* to *BPSL2312* in the *B. pseudomallei* K96243 genome) that was lacking from the genome of LMG 28154^T^, as well as from strains TSV85 and TSV86 (**Supplementary Table S2**). This difference explains the lack of nitrate reduction observed in phenotypic assays and might affect survival of strain LMG 28154^T^ under anaerobic conditions. Components of the cluster 1 Type VI secretion system (T6SS-1; *BPSS1493–BPSS1512*) are also missing in the LMG 28154^T^ genome. Knock-out mutants in *hcp1* (*BPSS1498*) are significantly less virulent in a Syrian hamster melioidosis model, with an LD_50_ more than 1000-fold higher than wild-type *B. pseudomallei* K96243 (Burtnick et al., 2011). The lack of this critical virulence factor might thus be responsible for attenuated pathogenicity of strain LMG 28154^T^. Finally, a putative *wza* and *wzc*-dependent exopolysaccharide gene cluster is encoded by genes *BSIN_4472–4480*, and does not have putative orthologs in the genomes of *B. thailandensis* E264^T^, *B. pseudomallei* K96243 and *B. oklahomensis* C6786^T^. The BSIN_4472–4480 genes are conserved with more than 95% identity at the nucleotide level in strains TSV85 and TSV86 (locus tags WS67_RS02485–02520 and WS68_RS24380–24415, respectively). Instead, predicted proteins of the gene cluster show high similarity, ranging from 45 to 70% identity, to a syntenic gene cluster of *B. cenocepacia* J2315^T^ (Supplementary Figure S5). *B. cenocepacia* produces several uncharacterized exopolysaccharides in addition to cepacian, the main exopolysaccharide of *B. cenocepacia* (Holden et al., 2009). However, some of these cryptic exopolysaccharide cluster genes are important for biofilm formation (Fazli et al., 2013), and the presence of this gene cluster in strain LMG 28154^T^ may explain in part its unusual mucoid appearance.

### Genome Wide AT Bias in Strain LMG 28154^T^

The average mol% G+C from *in silico* analysis of the strain LMG 28154^T^ genome sequence data was 64.34%. This value falls below the range of 65.7–68.5% for the genus *Burkholderia* as defined by Sawana et al. (2014) and within the 58.9–65.0% and 59.0–65.0% range for the newly described genera *Paraburkholderia* and *Caballeronia*, respectively (Dobritsa and Samadpour, 2016). The base composition of genomic sequences is highly variable across species and features prominently in descriptions of bacterial species (Tindall et al., 2010). It was one of two criteria (the other being the presence of conserved indels) that supported the first subdivision of the genus *Burkholderia* (Sawana et al., 2014), i.e., the separation of the species presently included in the genus *Burkholderia sensu stricto* from those are that presently classified in the genera *Paraburkholderia*, *Caballeronia*, and *Robbsia.* Because the presence of non-homologous regions with different average %G+C may explain the deviation of strain LMG 28154^T^ from related species, we measured the distribution of %G+C in 1 kb segments across the entire genome of representative *Burkholderia*, *Paraburkholderia*, *Caballeronia* and *Robbsia* species (**Figure 2**). The median %G+C of strain LMG 28154^T^ was 64.9%, indicating that extreme values are not responsible for the low average %G+C of the species. To estimate the %G+C on conserved sections of the genome, we also calculated the average %G+C of 2081 single-copy orthologous genes computed between strains LMG 28154^T^, *B. thailandensis* E264^T^, *B. pseudomallei* K96243, *B. oklahomensis* C6786^T^ and *B. glumae* BGR1 (Supplementary Figure S1). Overall, genes of LMG 28154^T^ displayed an average %G+C of 65.45%, below the averages of 67.74–68.81% for orthologs belonging to reference *Burkholderia* species. Moreover, the %G+C of orthologous genes belonging to strain LMG 28154^T^ were on average 2.53% lower than in *B. pseudomallei* K96243 (Paired Student’s *t*-test *p* < 0.001), 2.30% lower than in *B. thailandensis* E264^T^ (*p* < 0.001) and 3.36% lower than in *B. glumae* BGR1 (*p* < 0.001). This effect was exacerbated at the third codon positions, with a difference in average %G+C in LMG 28154^T^ compared to other species ranging from 6.15 to 7.81% (Supplementary Figure S1). Moreover, fourfold degenerate sites, which are not expected to be under selection for protein functionality, were significantly richer in AT in strain LMG 28254^T^ (Wilcoxon signed rank test *p* < 0.001) than in related species (Supplementary Figure S2). Similarly, the analysis of substitutions in a randomly chosen set of 17 intergenic regions revealed an AT mutational bias (ratio of GC > AT over AT > GC substitutions = 1.35, *n* = 87, data not shown). This value was significantly higher than the AT bias of 0.81 (binomial test *p* < 0.05) measured by Dillon et al. (2015). Together, these data are indicative of a pervasive, genome-wide AT-bias affecting strain LMG 28154^T^.

### Frequency of Recombination and Its Impact on GC-Biased Gene Conversion in Strain LMG 28154^T^

GC-biased gene conversion (gBGC), a process which drives the evolution of G+C content in mammals by favoring the conversion of GC-rich intermediates of recombination (Duret and Galtier, 2009), has recently been proposed to act in prokaryotes as well (Lassalle et al., 2015). We reasoned that if gBGC also influences average %G+C in *Burkholderia* species, species or genes that experience less recombination (for example due to ecological isolation) would have a lower average %G+C. We computed orthologous gene sets of strain LMG 28154^T^, *B. thailandensis* E264^T^, *B. pseudomallei* K96243, *B. oklahomensis* C6786^T^, *Burkholderia* sp. TSV86 and *B. glumae* BGR1 and calculated the probability of recombination for each ortholog set (see Materials and Methods) and evaluated if genes with high probability of recombination (*n* = 193) had higher %G+C than genes without (*n* = 1866). We did not find any statistically significant increase in average %G+C at the third codon position (which we showed above displayed the largest effect) in orthologous sets which showed evidence of recombination (Student’s *t*-test *p* = 0.387, data not shown). This is in accordance with the recent results of Lassalle et al. (2015) who did not find evidence that recombination was affecting %G+C of core genes of some species of the *B. cepacia* complex and the *B. pseudomallei* complex, in contrast to most bacterial species. Our method likely underestimated the number of genes subject to recombination because of the small genome dataset and our focus on single copy orthologs conserved in all genomes. However, because recombination is rare (<2%) in the core genome of species of the *B. pseudomallei* complex (Lassalle et al., 2015), our statistical analysis gives sufficient power to reject the hypothesis that gBGC contributes to the low %G+C of strain LMG 28154^T^. The lower average %G+C of strain LMG 28154^T^ is therefore not a result of a lower frequency of recombination and its impact on gBGC.

### No Reductive Genome Evolution in Strain LMG 28154^T^

Pervasive GC > AT mutation bias is a hallmark of reductive genome evolution, a phenomenon affecting intracellular pathogens as well as obligate symbionts with reduced effective population sizes (Moran, 2002), including some candidate *Burkholderia* species that cluster into the genus *Caballeronia* (Carlier et al., 2016; Pinto-Carbo et al., 2016). Other signs of reductive genome evolution include a significantly smaller genome size (Moran, 2002), an abundance of pseudogenes and insertion elements (Andersson and Andersson, 2001) and relaxed purifying selection (Kuo et al., 2009) compared to free-living relatives. *B. pseudomallei*, *B. oklahomensis* and *B. thailandensis* are environmentally acquired pathogens with the ability to replicate intracellularly inside macrophages (Wand et al., 2011; Willcocks et al., 2016). This raises the possibility that strain LMG 28154^T^ represents a lineage that transitioned to an exclusive host-associated lifestyle, thereby suffering from transmission bottlenecks. The genome of strain LMG 28154^T^ is slightly smaller than that of immediate free-living relatives, but still within the range of free-living *Burkholderia* (**Figure 2**). We identified only 57 potential pseudogenes in the genome of strain LMG 28154^T^, which amount to a total of 42.2 kb (0.76% of the genome), for a total coding capacity of the genome of 85%, which was also within the range of free-living *Burkholderia* species. The genome encodes 49 predicted proteins belonging to the COG category X (mobilome), which includes phage-derived proteins, transposases and other mobilome components (Galperin et al., 2015). This is again less than what has been reported for *Caballeronia* species in intermediate stages of reductive genome evolution (Lackner et al., 2011; Carlier and Eberl, 2012; Carlier et al., 2016; Pinto-Carbo et al., 2016). The most universal (but not exclusive) sign of ongoing reductive genome evolution is a relaxation of purifying selection, which results from an increased level of genetic drift experienced by recurrent population bottlenecks (Kuo et al., 2009). We therefore measured the ratios of non-synonymous to synonymous mutations (*dN*/*dS* ratio) of a set of 2890 orthologous genes which did not show evidence of recombination between the strains LMG 28154^T^, TSV86, *B. oklahomensis* C6786^T^ and *B. pseudomallei* K96243, to estimate the levels of genetic drift experienced by strains of the first pair of species on one hand, and other species of the *B. pseudomallei* complex on the other hand. We chose these sets of genomes because they presented comparable levels of sequence divergence (*dS*) and represent species with similar ecology. The average genome-wide *dN*/*dS* ratio between strains LMG 28154^T^ and TSV86 (*dN*/*dS* = 0.076) is indicative of relatively strong purifying selection and is consistent with a free-living or facultative lifestyle (Novichkov et al., 2009). Moreover, the genome-wide distribution of *dN*/*dS* values of LMG 28154^T^ /TSV86 showed only a slight upward shift compared to ortholog pairs in *B. pseudomallei* K96243/*B. oklahomensis* C6786^T^ (average *dN*/*dS* = 0.065) (Supplementary Figure S3). This indicates that purifying selection is not drastically relaxed in the novel taxon lineage compared to the *B. pseudomallei* – *B. oklahomensis* lineage. An analysis including the pair *B. oklahomensis* C6786^T^ and *B. thailandensis* E264^T^ showed similar results with genome-wide *dN*/*dS* = 0.066. It is possible that %G+C changes and altered codon usage bias in the LMG 28154^T^ genome affects the rate of evolution of synonymous sites, and in this case, the *dN/dS* ratio reported for the pair of strains LMG 28154^T^/TSV86 would in fact overestimate the intensity of purifying selection. However, we argue that the small difference in codon preference observed is unlikely to have arisen from selection for optimal codon usage given the similar translation machinery (e.g., number of tRNAs) in the species compared. The lack of evidence for rampant pseudogenization or IS proliferation, together with effective purifying selection and high coding density lead us to conclude that reductive genome evolution is not responsible for the genome-wide AT-bias in strain LMG 28154^T^.

### Functions Related to DNA Repair and Metabolism in Strain LMG 28154^T^

Contrary to what has been documented for most bacterial species, Dillon et al. (2015) recently reported that the mutational landscape in *Burkholderia* species is biased toward AT > GC substitutions. Historically, differences in mutation patterns have been thought to be the primary reason for the variation in %G+C in bacterial genomes (Sueoka, 1961), although more recent evidence suggested that selective forces also influence nucleotide composition, including in *Burkholderia* species (Balbi et al., 2009; Hershberg and Petrov, 2010; Dillon et al., 2015). To identify functions related to DNA repair and metabolism that were missing or altered in strain LMG 28154^T^ compared to close relatives with high %G+C, we computed the core genome of strains *B. oklahomensis* C6786^T^, *B. pseudomallei* K96243 and *B. thailandensis* E264^T^, and compared the COG functional assignments to the proteome of strain LMG 28154^T^. We found only 2 genes (locus tags: *BPSL1022* and *BPSS0452*) with a COG functional assignment falling into the L category (DNA replication and repair) in the core genome of *B. pseudomallei* K96243 that did not have any putative orthologs in the genome of strain LMG 28154^T^. BPSL1022 possesses a GIY-YIG domain found in the endonuclease SLX1 family, but the function of this enzyme in DNA repair or metabolism is unknown. BPSS0452 is a putative DNA polymerase/3′-5′ exonuclease of the PolX family, which may play a role in the base excision repair (BER) pathway (Banos et al., 2008; Khairnar and Misra, 2009). BLASTp analysis reveals that close homologs of BPSS0452 are conserved in various *Burkholderia* species but are notably absent from *B. mallei*, *B. glumae* and *B. gladioli* strains (Supplementary Figure S4). These species all have average genomic %G+C > 68.5%, indicating that loss of the PolX function is unlikely to be solely responsible for the altered %G+C of strain LMG 28154^T^. However, *BSIN_0972*, which encodes a putative DNA-3-methyladenine glycosylase 1 of the TagI family, is a pseudogene in strain LMG 28154^T^ (and also in TSV86). TagI proteins catalyze the excision of alkylated adenine and guanidine bases from DNA as part of the BER pathway (Bjelland and Seeberg, 1987). Instead, *BSIN_4945* encodes a protein of the same superfamily (COG2818) with 47% identity to the product of *BSIN_0972*, but this gene has no close homologs in *Burkholderia* species outside of strains TSV85 and TSV86 (Supplementary Figure S4). This gene was possibly acquired via horizontal gene transfer, since the closest homologs in the NCBI nr database belong to bacteria only distantly related to *Burkholderia* (closest hit in the genome of *Cohnella* sp. OV330 with 78% identity at the protein level, followed by *Noviherbaspirillum massiliense* JC206 with 77% identity). Because the putative ortholog of BSIN_0972 still seems functional in strain TSV85, and orthologs of BSIN_4945 are present in strains LMG 28154^T^, TSV85 and TSV86, acquisition of the alternative DNA-3-methyladenine glycosylase 1 encoded by *BSIN_4945* preceded the mutation events leading to the loss of the ancestral *tag* gene in the novel taxon lineage. Altered regulation of components of the BER pathway, or different affinity for alkylated purines could perhaps explain a bias in DNA composition in these bacteria.

### The Subdivision of the Genus *Burkholderia sensu lato* into the Genera *Burkholderia sensu stricto*, *Paraburkholderia* and *Caballeronia* Is Not Based on Solid Biological Evidence

Comparative 16S rRNA gene sequence analysis provided a phylogenetic image of the genus *Burkholderia sensu lato* that consisted of several main lineages and species that represented unique lines of descent (Gyaneshwar et al., 2011; Suarez-Moreno et al., 2012; Estrada-de los Santos et al., 2013). Depoorter et al. (2016) recently presented a comprehensive overview of the main species clusters and single species representing unique lines of descent within this genus. Although the value of the 16S rRNA gene as a single-gene marker for bacterial phylogeny is unsurpassed, it is well-known that its resolution for resolving organismal phylogeny has several limitations (Dagan and Martin, 2006), and branching levels in 16S rRNA based trees of *Burkholderia* species are commonly not supported by high bootstrap values. Yet, Sawana et al. (2014) used 16S rRNA gene sequence divergence as the basis to reclassify a large number of *Burkholderia* species into the novel genus *Paraburkholderia.* Species retained in the genus *Burkholderia* were further characterized by a % G+C content of 65.7–68.5%, and shared six conserved sequence indels, while all other *Burkholderia* strains examined had a % G+C content of 61.4–65.0% and shared two conserved sequence indels; all the latter species were reclassified into the novel genus *Paraburkholderia.* Very rapidly, the classification of *Paraburkholderia* species was revisited by Dobritsa and Samadpour (2016) and part of the *Paraburkholderia* species were further reclassified into the novel genus *Caballeronia.* The latter species clustered again together in a 16S rRNA based phylogenetic tree and shared five conserved sequence indels. More recently, *Paraburkholderia andropogonis*, one of the species that consistently forms a unique line of 16S rRNA descent, was further reclassified into the novel genus *Robbsia*; the G+C content of its type strain is 58.92 mol % (Lopes-Santos et al., 2017).

The availability of numerous whole-genome sequences nowadays presents ample opportunities for phylogenomic studies to generate multi-gene based phylogenetic trees with superior stability and that better reflect organismal phylogeny (Yutin et al., 2012; Wang and Wu, 2013). However, it has been argued that bootstrap and similar support values increase with the increasing number of sites sampled (Phillips et al., 2004) such that a high bootstrap proportion for a multi-gene concatenated phylogeny does not necessarily mean that the tree is thus likely to be correct (Thiergart et al., 2014). The phylogenomic tree presented in the present study (**Figure 2**) is based on 15092 conserved nucleotide positions from 21 nearly universal, single copy gene markers and shows that *Caballeronia* and *Paraburkholderia* species represent a single, mixed and heterogeneous lineage, as do species retained in the genus *Burkholderia sensu stricto*. In contrast, *R. andropogonis*, the sole member of the genus *Robbsia*, continued to form a well-separated branch below the *Burkholderia- Paraburkholderia- Caballeronia* cluster (**Figure 2**). We obtained very similar results earlier (Depoorter et al., 2016) through analysis of 53 ribosomal protein-encoding genes (Jolley et al., 2012), while a recent phylogenomic study based on the analysis of 106 conserved protein sequences revealed distinct and stable clusters for each of the genera *Burkholderia sensu stricto*, *Caballeronia* and *Paraburkholderia* (Beukes et al., 2017).

Although G+C content long appeared to discriminate between *Burkholderia sensu stricto* and other *Burkholderia* species, the data presented above for strain LMG 28154^T^ demonstrated that there is an overlapping continuum in G+C content of *Burkholderia sensu stricto* species and other species previously classified within this genus. Finally, the sole remaining criterion that discriminated between *Burkholderia sensu stricto* and other *Burkholderia* species, is the presence of six conserved sequence indels (Sawana et al., 2014). *Burkholderia* are multi-chromosome bacteria and the latter six genes are located on chromosome 2 in most species. The latter chromosome is highly dynamic in *Burkholderia* species (Cooper et al., 2010) and none of the genes (i.e., BCAM2774, BCAM0964, BCAM0057, BCAM0941, BCAM2347, and BCAM2389) containing the indels appeared essential for growth on either rich or minimal media (Wong et al., 2016). Together, these data demonstrate that there is no solid biological evidence for the ongoing taxonomic dissection of the genus *Burkholderia*. In our opinion, the proposals of the genera *Caballeronia* and *Paraburkholderia* are ill-founded. Researchers, referees and editors of scientific journals should be aware that the rules of bacterial nomenclature stipulate that names that were validly published remain valid, regardless of subsequent reclassifications (Parker et al., 2017). Authors can therefore continue to work with the original (*Burkholderia*) species names as these were all validly published, and focus their efforts on the understanding of the biology of these fascinating bacteria.

## Conclusion

The genotypic and phenotypic distinctiveness of strain LMG 28154^T^ warrants its classification as a novel species within the *B. pseudomallei* complex (Depoorter et al., 2016; Price et al., 2016) of the genus *Burkholderia. B. cepacia* complex MLST analysis revealed that the isolates R-20802 and R-50762 have the same allelic profile (ST-813) as strain LMG 28154^T^, and therefore represent the same strain that persisted in this Canadian CF patient. Strain LMG 28155 represents a second sequence type (ST-815) that differs in six out of seven alleles (i.e., in 17 nucleotides of a total of 2773 nucleotide positions) examined. Finally, extraction of the MLST loci from the genome sequence of strain TSV85 and the ANI values discussed above (**Supplementary Table S1**), demonstrated that it represents a third ST, i.e., ST-1295 that also differs in six out of seven alleles from the other STs in this species. This novel species has been recovered from human or environmental samples on three continents. It has a unique MALDI-TOF MS profile and displays several differential phenotypic characteristics that will facilitate its identification in diagnostic laboratories. We propose to formally classify this novel species as *B. singularis* sp. nov. with strain LMG 28154^T^ as the type strain. The same strain was recently isolated again from the same patient (data not shown) but colonies unexpectedly were non-mucoid on Columbia sheep blood agar. Together, this type strain has been isolated from the same Canadian CF patient over the course of 14 years, indicating that in this case at least, the clinical impact of this species has been relatively mild.

### Description of *Burkholderia singularis* sp. nov.

#### *Burkholderia singularis* (sin.gu’la.ris. L. adj. *singularis*, singular, remarkable, unusual)

Cells are Gram-negative, non-sporulating rods. All strains grow on Columbia sheep blood agar, *B. cepacia* Selective agar, Yeast Extract Mannitol agar and MacConkey agar. Mucoid growth and no hemolysis on Columbia sheep blood agar. Growth is observed at 42°C. Motility is strain dependent. No pigment production. Oxidase activity is slowly positive. No lysine or ornithine decarboxylase activity. Activity of β-galactosidase is present but no nitrate reduction (both as determined using the API 20NE microtest system). Gelatin liquefaction and esculin hydrolysis are strain-dependent. Acidification of glucose, maltose, lactose and xylose but not sucrose; strain-dependent reactions for acidification of adonitol. The following fatty acids are present in major amounts: C_16:0_, C_16:0_ 3-OH, C_18:1_ ω7c and summed features 2 and 3; C_14:0_, C_16:0_ 2-OH, C_16:1_ 2-OH, C_17:0_ cyclo, and C_18:1_ 2OH are present in moderate amounts. Strains in the present study have been isolated from human respiratory specimens and a water sample.

The type strain is LMG 28154^T^ (= CCUG 65685^T^). It does not liquefy gelatin, hydrolyse esculin and is non-motile; it acidifies adonitol. Other phenotypic characteristics are as described for the species. Its G+C content is 64.34%.

## Accession Numbers

The GenBank/EMBL/DDBJ accession numbers for the 16S rRNA gene sequences of strains LMG 28154^T^ and LMG 28155 are LK023501 and LT717095, respectively. The accession numbers for the LMG 28154^T^ genome are FXAN01000001-FXAN01000135.

## Author Contributions

PV and AC conceived the study and wrote the manuscript. JZ, MM, JW, and BC contributed to conceptualization. AC, EP, and DS carried out the genomic data analyses. CP and BDS analyzed the 16S rRNA and MLST data and performed phylogenetic analyses. CP, BDS, EP, DS, DH, TH, and AB carried out wet-lab microbiological analyses. PV, JZ, MM, JW, and BC generated the required funding. All authors read and approved the final manuscript.

## Conflict of Interest Statement

The authors declare that the research was conducted in the absence of any commercial or financial relationships that could be construed as a potential conflict of interest.

## Footnotes

1 http://www.bacterio.cict.fr/

2 http://www.arb-silva.de/aligner/

3 https://pubmlst.org/bcc/

4 http://pubmlst.org/bpseudomallei

5 http://www.sanger.ac.uk/science/tools/smalt-0

6 http://www.ebi.ac.uk/ena

## Supplementary Material

**TABLE S1 |** ANI values between strain LMG 28154^T^ and phylogenetically neighboring species.

**TABLE S2 |** Functions from the core genome of strains *B. thailandensis* E264^T^, *B. pseudomallei* K96243 and *B. oklahomensis* C6786^T^ lacking from the genome of LMG 28154^T^.
