# Supplementary material for: Comparative Genomics of Burkholderia singularis sp. nov., a Low G+C Content, Free-Living Bacterium That Defies Taxonomic Dissection of the Genus Burkholderia
Source: Front Microbiol. 2017 Sep 6;8:1679. doi: 10.3389/fmicb.2017.01679 (PMC5592201; doi:10.3389/fmicb.2017.01679)
Supplement: Supplementary file 3 [file Image_1.PDF]

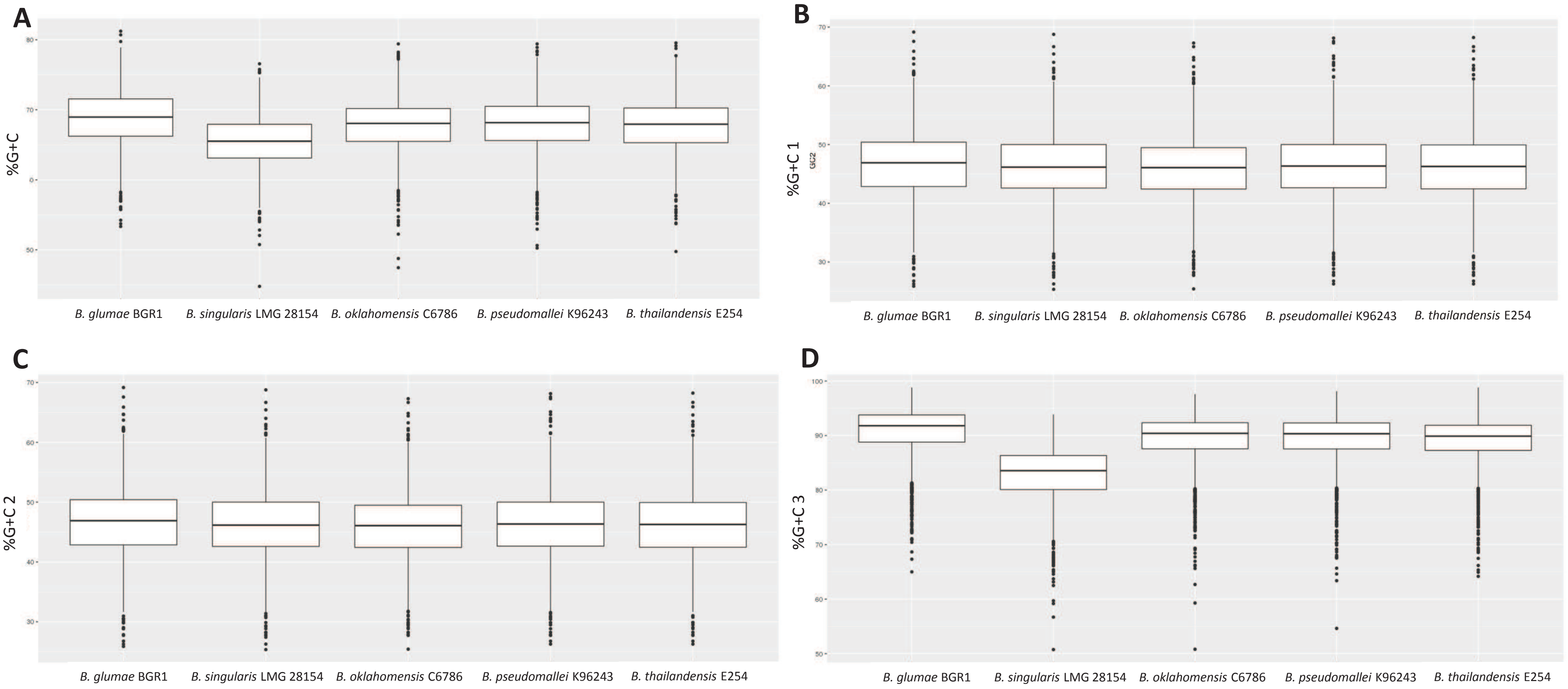

Figure S1. Distribution % G+C across orthologous gene sets. A. Box plot showing average % G+C in single-copy orthologous genes across the genomes of *B. singularis* LMG 28154^{T} and neighboring *Burkholderia* species (n = 2081). B. Average % G+C at the first codon position. C. Average % G+C at the second codon position. D. Average % G+C at the third codon position.

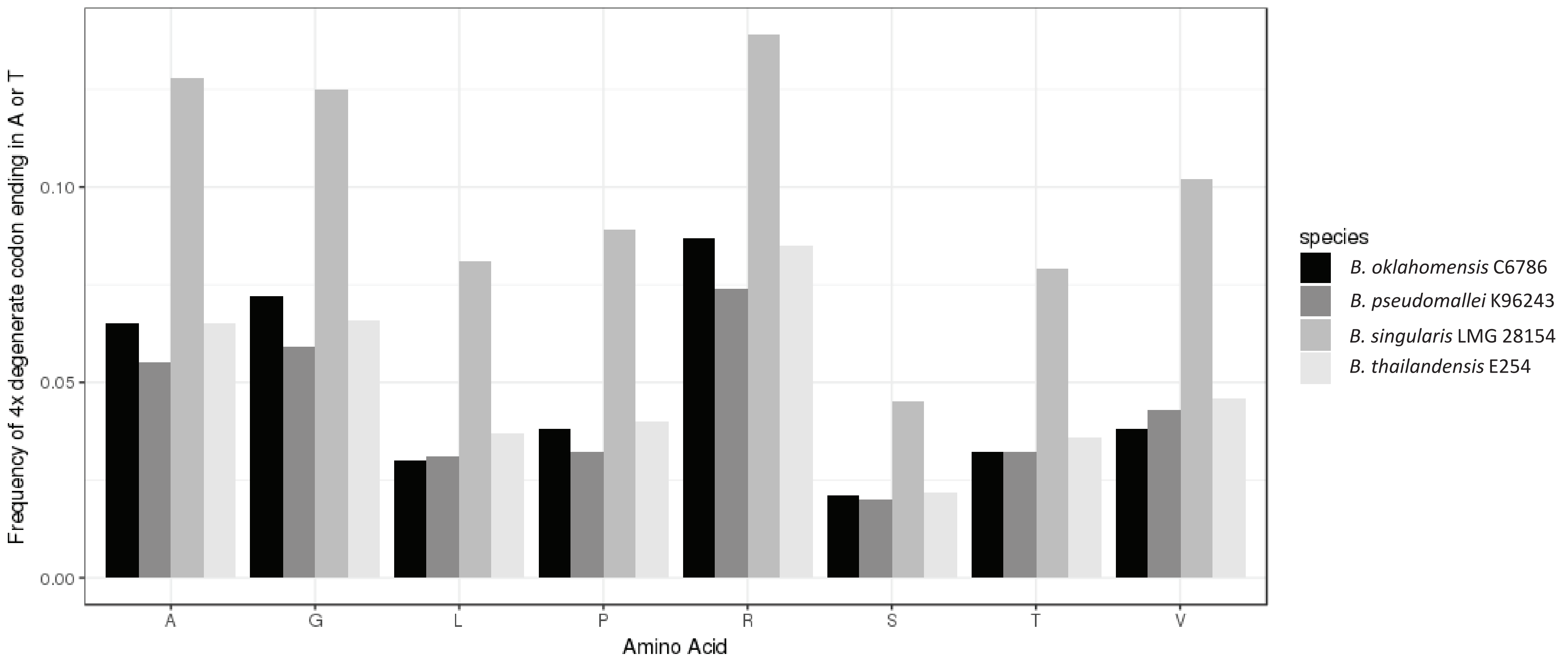

Figure S2. Frequency of AT bases at four-fold degenerate codon positions of 2081 single copy orthologous genes across the genomes of *B. singularis* LMG 28154^{T} and neighboring *Burkholderia* species.

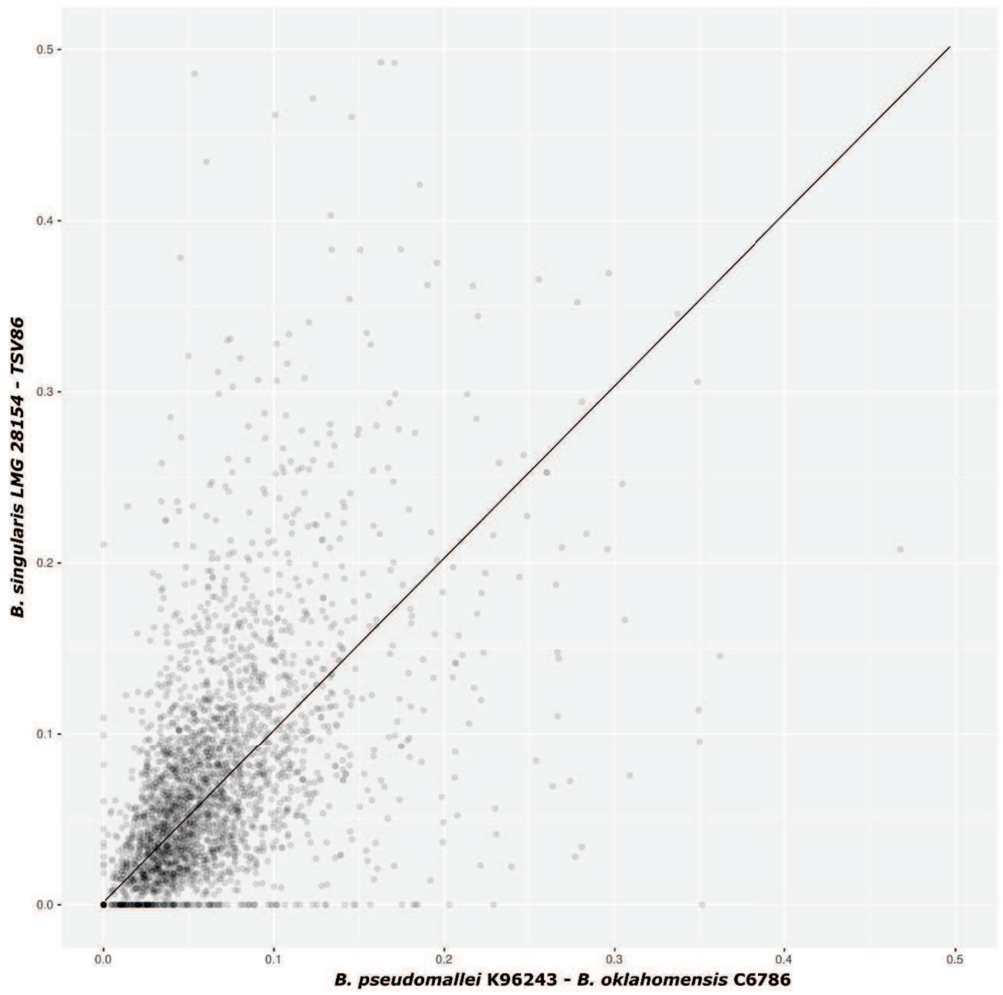

Figure S3. Comparison of selective constraint (*dN*/*dS*) in the *B. singularis* versus *B. pseudomallei* complex lineages. Each point represents the *dN*/*dS* value for each ortholog from a dataset comprising single copy orthologs that do not show evidence of recombination and filtered for 0.1< *dS* < 1 (n = 2486).

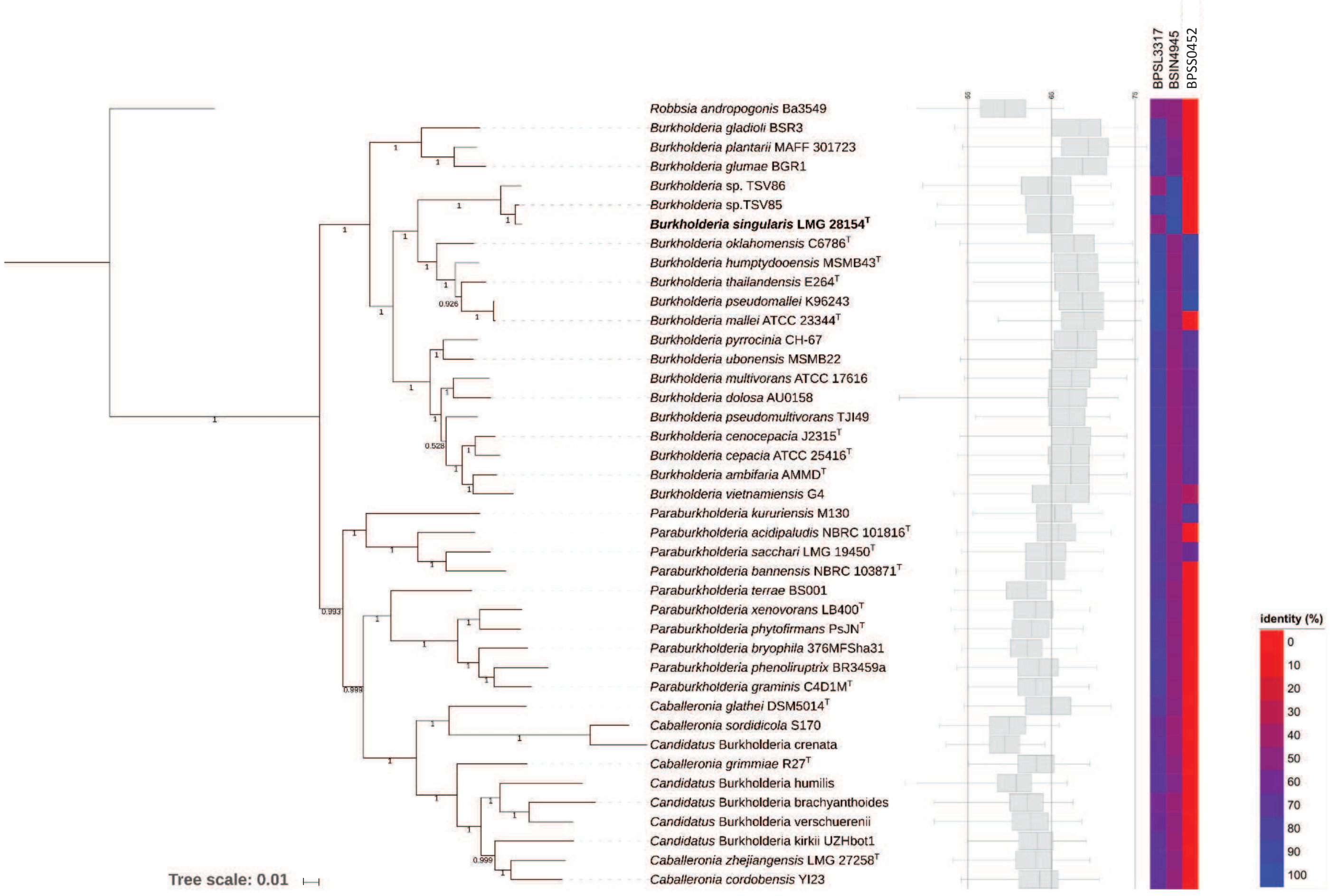

Figure S4. Distribution of selected genes with a predicted role in DNA repair in representative *Burkholderia* species. See main text and Figure 2 for details about phylogenetic and % G+C analyses. The heatmap plot shows the average identity of the best Blastp hit in the genome using as query the predicted AA sequences of genes BPSL3317 (*B. pseudomallei* K96243), BPSS0452 (*B. pseudomallei* K96243) and BSIN_4945 (*B. singularis* LMG 28154).

A

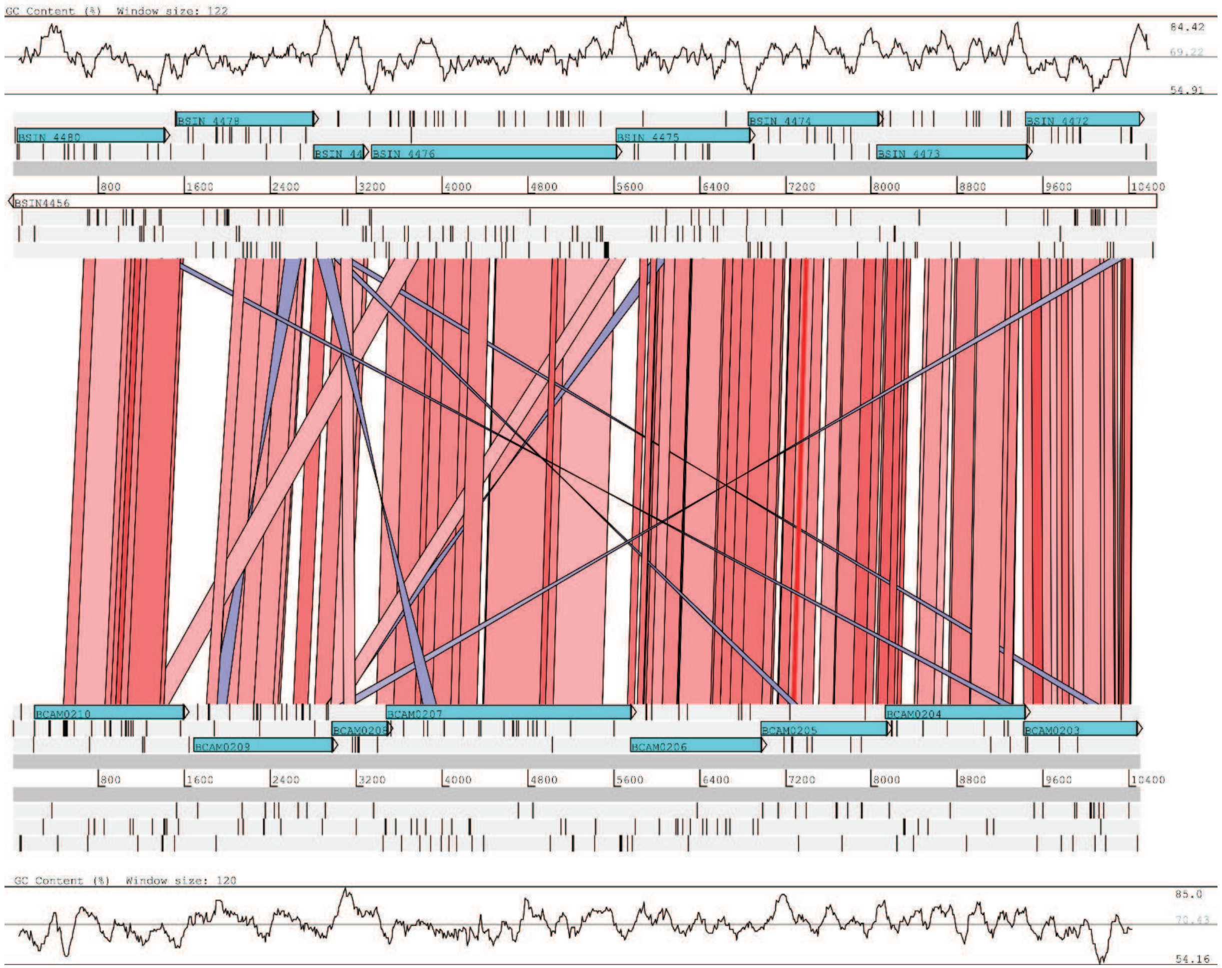

B

Locus tag B. singularis LMG 28154T	Locus tag B. cenocepacia J2315	Pairwise identity (Blastp)	Putative product
BSIN_4480	BCAM0210	51%	Undecaprenyl-phosphate galactose phosphotransferase
BSIN_4478	BCAM0209	45%	polysaccharide export lipoprotein Wza
BSIN_4477	BCAM0208	60%	Low molecular weight protein-tyrosine phosphatase Wzb
BSIN_4476	BCAM0207	54%	Tyrosine protein kinase Wzc
BSIN_4475	BCAM0206	56%	Glycosyltransferase
BSIN_4474	BCAM0205	67%	Uncharacterized conserved protein
BSIN_4473	BCAM0204	70%	Permease of the major facilitator superfamily
BSIN_4472	BCAM0203	51%	L-lactate permease

Figure S5: Comparison of putative exopolysaccharide gene clusters of *B. singularis* LMG 28154^{T} and *B. cenocepacia* J2315. **A**. Sequence alignment of the putative EPS gene cluster of *B. singularis* LMG 28154T (top) and *B. cenocepacia* J2315 (bottom). Red connecting lines depict tblastx matches (evalue < 10^{-3}) in the forward strand as computed on the WebACT web service (http://www.webact.org/WebACT/home), blue connecting lines indicate matches on the reverse strand. Graphs at the top and bottom show the %G+C (average over a 120 bp window) **B**. Summary of annotated features of the *B. singularis* LMG 28154^{T} putative EPS gene cluster and corresponding annotations in *B. cenocepacia* J2315. Pairwise identity scores were calculated using Blastp (evalue < 10^{-3}).
